# On Extracting Digitized Spiral Dynamics’ Representations: A Study on Transfer Learning for Early Alzheimer’s Detection

**DOI:** 10.3390/bioengineering9080375

**Published:** 2022-08-09

**Authors:** Daniela Carfora, Suyeon Kim, Nesma Houmani, Sonia Garcia-Salicetti, Anne-Sophie Rigaud

**Affiliations:** 1 SAMOVAR, Télécom SudParis, Institut Polytechnique de Paris, 9 rue Charles Fourier, CEDEX, 91011 Evry, France; 2 AP-HP, Groupe Hospitalier Cochin Paris Centre, Pôle Gérontologie, Hôpital Broca, 75013 Paris, France; 3 EA 4468, Paris Descartes University, 75006 Paris, France

**Keywords:** online spiral analysis, automatic feature extraction, transfer learning, Alzheimer’s disease, classification

## Abstract

This work proposes a decision-aid tool for detecting Alzheimer’s disease (AD) at an early stage, based on the Archimedes spiral, executed on a Wacom digitizer. Our work assesses the potential of the task as a dynamic gesture and defines the most pertinent methodology for exploiting transfer learning to compensate for sparse data. We embed directly in spiral trajectory images, kinematic time functions. With transfer learning, we perform automatic feature extraction on such images. Experiments on 30 AD patients and 45 healthy controls (HC) show that the extracted features allow a significant improvement in sensitivity and accuracy, compared to raw images. We study at which level of the deep network features have the highest discriminant capabilities. Results show that intermediate-level features are the best for our specific task. Decision fusion of experts trained on such descriptors outperforms low-level fusion of hybrid images. When fusing decisions of classifiers trained on the best features, from pressure, altitude, and velocity images, we obtain 84% of sensitivity and 81.5% of accuracy, achieving an absolute improvement of 22% in sensitivity and 7% in accuracy. We demonstrate the potential of the spiral task for AD detection and give a complete methodology based on off-the-shelf features.

## 1. Introduction

The analysis of online handwritten tasks has emerged these last years as a pertinent behavioral modality to aid clinicians in the diagnosis of neurodegenerative pathologies. Indeed, digital tablets allow capturing handwritten traces as spatiotemporal signals (“online” signals) that carry fine dynamic information about the writing process. Recent surveys in the field demonstrate the importance of this noninvasive modality to assess the health status of a person, by providing additional information to cognitive tests [1,2,3].

Initiated with memory loss, Alzheimer’s disease (AD) impairs motor functions progressively. Fine motor skills acquired in early life decline as the disease advances. In the literature, additionally to handwritten tasks with semantic content, different elementary graphic tasks have been analyzed to extract AD markers: straight lines in different orientations [4,5] that require coordination of fingers and wrist, connected cursive loops [6], and concentric superimposed circles [7]. On the other hand, another graphic task, Archimedes spiral, was proposed by Pullman [8] to assess tremors on a digitizer. Interestingly, the spiral task has been intensively studied for Parkinson’s disease (PD) [9,10,11,12,13] and Essential Tremor detection [14,15], but hardly for AD. In [9,13], the spiral task was analyzed in a standalone mode for PD detection; but more recently, it has also been fused to other tasks (such as handwritten text, isolated words, and bigrams) in works exploiting deep learning [16,17,18,19,20,21]. In [16,18], we note a significantly higher accuracy with the spiral task compared to other tasks on the same population. This result emphasizes that the spiral task is particularly well suited for decision aid to clinicians in detecting motor dysfunction related to PD. Additionally, those works analyzing the spiral task in a standalone manner also point out its capability of characterizing stages of the disease that are difficult to detect with some standard clinical tests [10].

For these reasons, in this paper, we focus on the analysis of Archimedes’ spiral for early-stage AD detection. We exploit deep learning (DL) techniques to extract automatically features on spiral images generated from online sequences.

### 1.1. Positioning with Regard to the State-of-the-Art in Deep Learning

Two main trends emerge in the literature addressing neurodegenerative diseases’ detection with DL techniques on handwritten graphical tasks. First, works exploiting transfer learning for high-level feature extraction; such works use images generated from online handwriting [16,21,22] while keeping the shape of the pen trajectory and assessing the health status of the subject with another classifier. Second, we find works that train DL classifiers on time series-based images [17,18,20], which do not convey information about the shape of the pen trajectory. More precisely, in [17,18], raw six-dimensional time series are captured with a smart pen that contains six sensors. Such time series or temporal functions are converted into a grey-level image, in which rows are time in milliseconds and columns represent the six time series. In [20], spectrogram images are constructed in grey level for each time series and given as input to the DL model. We note that the approaches in [16,17,18,20] do not allow taking advantage simultaneously of visual content and dynamic information of the drawing. In [21], the authors propose to include, in pen trajectory images, in-air trajectories and to some extent local velocity, in two ways: the first is binary, since the in-air trajectory, displayed in grey, encodes the presence of a pen-up in the drawing (pen-downs remain in black). Second, to encode pen velocity, the sampled points of the spiral trajectory are not connected: as the sampling rate of the digitizer is fixed, when the pen moves faster on the writing surface, the spacing between consecutive captured points increases, and accordingly, decreases when the pen moves slower. Thus, velocity is encoded through points’ spacing on the image of the handwriting, instead of an explicit value belonging to a given discrete range. This work shows two main drawbacks: first, it proposes a simultaneous encoding of velocity and pen-ups in the same handwriting image, which makes it difficult to analyze separately the impact of each dynamic information (pen-ups’ occurrence and changes in velocity) for pathology detection. Second, the dynamic range of any other parameter, such as pen pressure, pen inclination, or higher order derivatives of pen position, is difficult to exploit through this pure spatial encoding on the pen trajectory image.

In this context, we propose a novel framework to embed dynamic information in spiral images generated from online sequences, for enhancing discrimination between HC and AD populations. This framework allows taking advantage simultaneously of static and dynamic characteristics of the spiral drawing. Our approach proposes instead to embed each dynamic parameter in a devoted spiral image, explicitly, as a value in the [0, 255] interval, in greyscale. Contrary to the above-mentioned works [17,18,21], our approach allows assessing the impact of each dynamic parameter separately, as a particular “view” of the drawn spiral; we are thus able to study which dynamic information is the most pertinent for discriminating between AD and HC subjects.

By exploiting transfer learning, we compare off-the-shelf automatically extracted features, at different levels of representation, in terms of their capability to discriminate between AD and HC populations. In [16,21,22], deep representations are extracted at the highest layer before the fully connected ones, and later fed to classifiers. To our knowledge, within the field of neurodegenerative diseases’ detection through handwritten graphical tasks analysis, our work is the first to carry out a comprehensive study using representations extracted at lower layers of the deep network, especially in terms of sensitivity and specificity metrics. This trend in transfer learning is of high potential, as shown in other image processing tasks [23]: according to the field, very deep networks may indeed provide high-quality off-the-shelf features in early layers. In the area of neurodegenerative diseases’ assessment, transfer learning is of particular interest: most of the time, very little data is available; the heterogeneity between subjects is important, especially at an early stage of the disease; finally, it avoids the strong difficulty of tailoring by hand features to a population. AlexNet [24] has proven its capacity to extract pertinent features on static images of handwriting at a high level (fc7 layer) [16]. One main objective of the present work is to study how our novel approach to embed dynamic parameters impacts the quality of representations extracted from AlexNet at lower or intermediate layers.

To enhance pathology detection, usually, experts (classifiers) are trained on different handwritten tasks and cooperate through decision fusion [16,17,18,20,21]. Thanks to our approach providing for each dynamic parameter a specific “view” of the spiral drawing, our work investigates, through different fusion frameworks, which are the most pertinent dynamic information discriminating AD and HC populations, based on only one task.

An overview of the main contributions of this work is presented in the next subsection.

### 1.2. Overview of Our Contributions

Our study clarifies the potential of the spiral task as a dynamic gesture for early AD detection and defines the most pertinent methodology for exploiting transfer learning in this context. More precisely, in this work, we propose:

(i) A novel embedding of dynamic parameters, each encoded separately in different images of a same spiral, providing different “views” of the spiral image (different types of “hybrid“ images);
(ii) A comprehensive study on which dynamic parameters are more discriminant at the classification step, for AD detection. Indeed, we study in this framework of transfer learning different dynamic parameters never studied before in the literature;
(iii) A comparative study on features automatically extracted at different levels of representation by transfer learning; this study is carried out on a reference architecture in the area of neurodegenerative diseases’ assessment, namely AlexNet [24];
(iv) A complete study on the scope of fusion. Our approach allows new possibilities for fusing dynamic information: at input images (low-level fusion), at the feature level (on representations extracted with AlexNet), and at the experts’ decision level.

### 1.3. Organization of the Paper

This paper is organized as follows: in Section 2, we detail the database, its associated acquisition protocol, and present our methodology for analyzing dynamically enriched spiral trajectory images through transfer learning. Section 3 describes our experimental setup and the obtained results. Section 4 states our conclusions and perspectives for future work.

## 2. Materials and Methods

### 2.1. Data Acquisition Protocol

Our private dataset was acquired at Broca Hospital in Paris, in the framework of the ALWRITE project [25,26], a French research project on handwriting analysis for early AD assessment. This study was approved by the Ethics Committee “Ile-de-France III” of APHP (Assistance Publique Hôpitaux de Paris). All participants freely signed a consent form after receiving information on the study. Our database contains one spiral for each of the 75 participants captured in one session, namely 30 early-stage AD patients and 45 HC subjects with a mean age of 80.2 ± 8.8 and 73.5 ± 6.1, respectively. AD patients were diagnosed based on DSM-5 criteria [27] and considered as having early-stage AD if their MMSE was over 20. On the other hand, HC performed neuropsychological tests to ensure their cognitive profile is normal. Participants with medical problems such as stroke and other neurodegenerative diseases were not included. The average MMSE is 22.1 ± 4 for AD patients and 29 ± 0.98 for HC.

**Figure 1:**
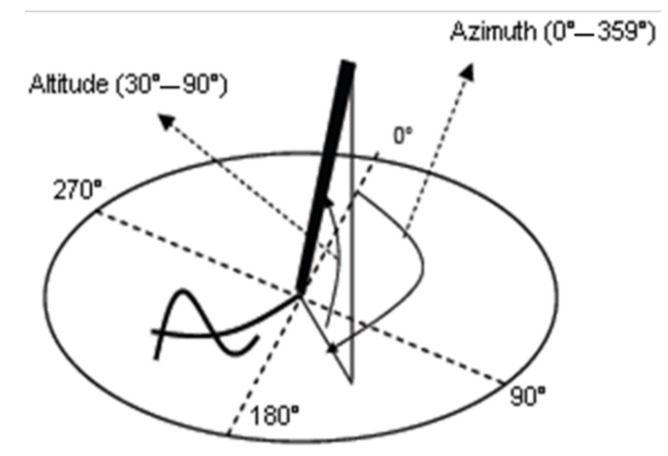
Azimuth and altitude angles captured by the Wacom digitizing tablet.

The whole protocol contained one task consisting in drawing one Archimedes’ spiral along with some other graphic tasks. Participants drew the spiral with an inking pen on a sheet of paper put on top of the tablet Wacom Intuos Pro Large. The digitizing tablet sampled the pen trajectory at 125 Hz and captured 5 raw temporal functions: pen position (*x*(*t*), *y*(*t*)), pen pressure *p*(*t*), and two pen inclination angles, namely azimuth *Az*(*t*) and altitude *Alt*(*t*)*,* as shown in Figure 1.

**Figure 2:**
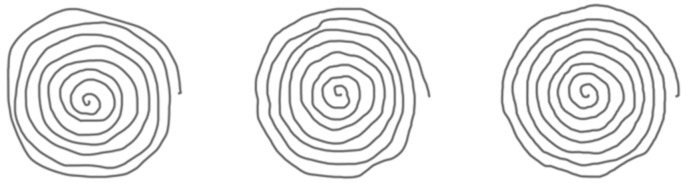
HC raw (pen-down) spiral trajectory images generated from coordinate sequences.

**Figure 3:**
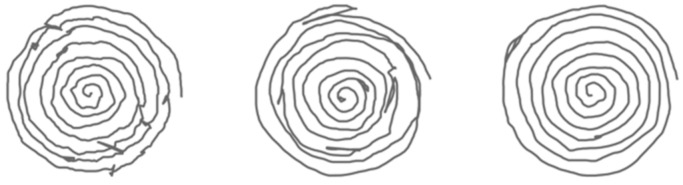
AD raw (pen-down) spiral trajectory images generated from coordinate sequences.

The Wacom tablet also captures the in-air trajectory of the pen (pen-ups) up to 1 cm off the tablet surface. Examples of raw spirals from HC and AD patients are displayed in Figure 2 and Figure 3, respectively, when considering only pen-down (on paper) trajectories.

### 2.2. Methodology for the Analysis of Archimedes Spiral Images

In this section, we first present how we embed dynamic information in spiral trajectory images. Second, we describe the feature extraction process by the deep neural model through transfer learning. Finally, we present the complete system architecture and evaluation setup for discriminating between AD and HC populations.

#### 2.2.1. Embedding Dynamics in Trajectory Images

We generate the spiral’s trajectory image from the online sequence of pen coordinates captured by the digitizer. This results in the images shown in Figure 2 and Figure 3 when displaying only pen-down trajectories (when the pen touches the tablet surface).

In this study, we consider different dynamic information: pen pressure, pen altitude, pen velocity, and pen acceleration. We compute pen velocity and pen acceleration at each point of the trajectory, according to Equations (1) and (2).

(1) $$v\left(t\right)=\sqrt{v_{x}^{2\;}\left(t\right)+v_{y}^{2\;}\left(t\right)}$$

where $v_{x}\left(t\right)=\frac{x\left(t+1\right)-x\left(t\right)}{\Delta t}$ and $v_{y}\left(t\right)=\frac{y\left(t+1\right)-y\left(t\right)}{\Delta t}$.

(2) $$a\left(t\right)=\sqrt{a_{x}^{2\;}\left(t\right)+a_{y}^{2\;}\left(t\right)}$$

where $a_{x}\left(t\right)=\frac{v_{x}\left(t+1\right)-v_{x}\left(t\right)}{\Delta t}$ and $a_{y}\left(t\right)=\frac{v_{y}\left(t+1\right)-v_{y}\left(t\right)}{\Delta t}$.

We also consider air-trajectory points (pen-ups) to take into account the whole gesture during the task’s execution. Indeed, in [15] the authors show that in-air trajectories may contribute to a better detection of pathological behaviors during handwritten tasks.

Such dynamic parameters are embedded in the spiral trajectory image in a pointwise manner: a min-max normalization is performed, considering the minimum and maximum values of such parameters on the whole population. The resulting normalized values that belong to the [0, 1] interval, are each mapped to a grayscale level in the interval [0, 255]. This normalization process makes comparable images of different persons on the same scale.

**Figure 4:**
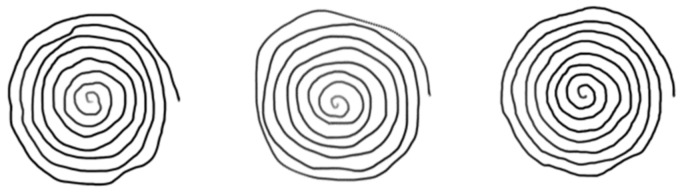
HC hybrid spiral images embedding pointwise pressure values in grayscale. Low and high-pressure levels on pen-down trajectories are displayed from white to black, respectively.

**Figure 5:**
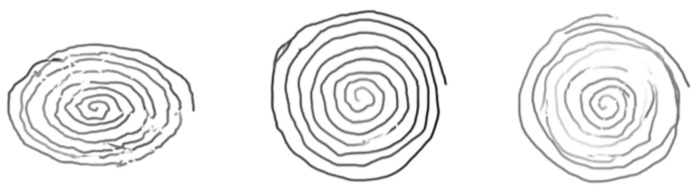
AD hybrid spiral images embedding pointwise pressure values in grayscale. Low and high-pressure levels on pen-down trajectories are displayed from white to black, respectively.

**Figure 6:**
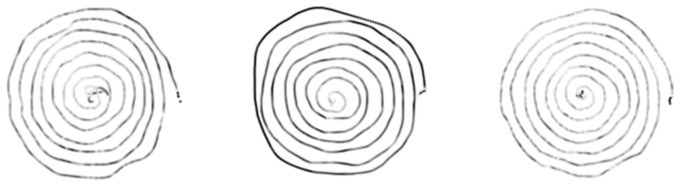
HC hybrid spiral images embedding pointwise velocity values in grayscale. Low and high-velocity values on both pen-down and pen-up trajectories are displayed from white to black, respectively.

**Figure 7:**
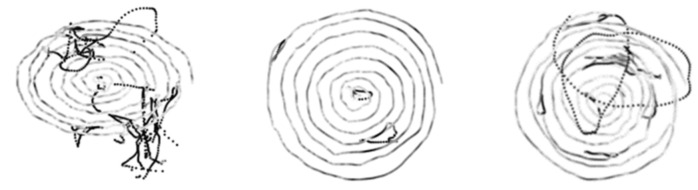
AD hybrid spiral images embedding pointwise velocity values in grayscale. Low and high-velocity values on both pen-down and pen-up trajectories are displayed from white to black, respectively.

Figure 4 and Figure 5 display examples of images embedding pressure information (referred to in the following as “hybrid” pressure images). Figure 6 and Figure 7 show images of the same persons of Figure 4 and Figure 5, embedding velocity information (referred to in the following as “hybrid” velocity images).

In hybrid velocity images of AD patients shown in Figure 7, we observe that the in-air trajectory points (pen-ups) show higher velocity values than on-paper trajectory points (pen-downs). In Figure 5, the corresponding hybrid pressure images show “broken” trajectories when pen-ups appear during the task.

On the HC population, hybrid pressure images (Figure 4) show rather high-pressure values on pen-downs; the corresponding hybrid velocity images (Figure 6) show accordingly rather slow trajectories on pen-downs.

In the next section, we describe feature extraction with transfer learning, when such hybrid spiral images embedding dynamic information are given as input to AlexNet [24] deep network.

#### 2.2.2. Automatic Feature Extraction with Transfer Learning

We consider in total four hybrid types (or “views”) of spiral images, including pressure, velocity, altitude, and acceleration dynamics. In this way, AlexNet extracts automatically a feature representation vector and that for each type of hybrid image.

**Figure 8:**
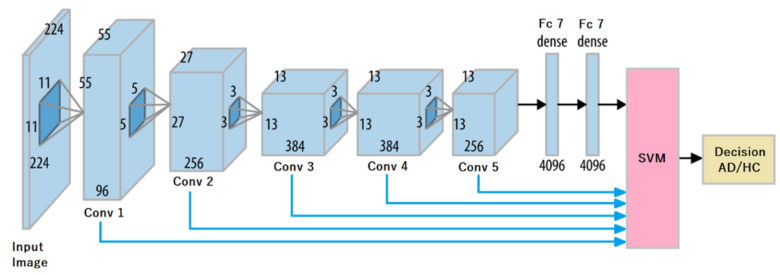
Feature extraction at different layers of AlexNet.

In this work, we study the influence of feature representations extracted at different layers of AlexNet (see Figure 8). To our knowledge, there is no existing study ensuring that *fc7* representations are the most effective for performing transfer learning for our specific classification problem. Besides, in the very prolific literature on DL, we can note that according to the type of input given to a convolutional neural network, the proper level for feature extraction may vary [23]. Interestingly, middle layers may convey better features according to the nature of the classification problem, as shown in [23]. For this reason, to investigate the representation capability of each layer for our classification task, we consider the output of each layer as a feature descriptor, and report the corresponding performance with a support vector machine (SVM) classifier, as illustrated in Figure 8.

#### 2.2.3. Support Vector Machine (SVM) Classification

According to each type of hybrid image given as input to AlexNet (pressure, velocity, altitude, acceleration), a corresponding SVM expert is trained on a feature vector extracted at a given layer. We compare the classification performance of each SVM expert in terms of the type of hybrid image, but also in terms of the layer at which off-the-shelf AlexNet features are extracted.

We consider an SVM with an RBF kernel in order to manage the high dimension of the feature representations (vectors) extracted from AlexNet. Note that the dimension of the feature vector differs from one layer to another (see Figure 8).

As we have at disposal one spiral per person, for 45 HC and 30 AD patients, we perform 10 random samplings of 30 HC among the 45 HC, in order to have the same number of samples per class when training the SVM classifier. In each sampling, we train the SVM on 20 HC and 20 AD and test on the remaining 10 HC and 10 AD. Average performance is given in terms of accuracy, specificity (percentage of HC well classified), and sensitivity (percentage of AD patients well classified), considering the 10 samplings, along with their standard deviations.

We applied a grid search optimization of the SVM parameters by performing a 5-fold cross-validation of the training data (each sampling of 20 HC and 20 AD). The best configuration on average on the validation dataset (one-fifth of 20 HC and 20 AD) is used to assess the SVM performance on the test dataset (the remaining 10 HC and 10 AD).

In order to enhance the discrimination between HC and AD populations, we combine the decisions of different SVM classifiers by majority voting; note that each SVM is an expert of a specific type of dynamic information.

## 3. Experimental Results

### 3.1. Extracting Off-the-Shelf Features at Different Layers

#### 3.1.1. Pressure-Based Hybrid Images

Table 1 displays results obtained on raw images, displaying only pen-down trajectories. We note that *fc7* gives the best accuracy compared to other layers. Nevertheless, the sensitivity is low (62%) and unbalanced with the specificity (87%).

Instead, for pressure-based hybrid images, as shown in Table 2, one of the best results is obtained when considering *Conv5* descriptors. Comparatively to raw spiral images (Table 1), we obtain in this case an improvement in accuracy, reaching 79.5%, and striking a better balance between specificity and sensitivity measures. We note in particular the strong improvement of sensitivity, from 62% with the raw image, to 77% with pressure encoding on the spiral trajectory.

Interestingly, we notice in Table 2 that *Conv3* descriptors achieve an almost equivalent sensitivity (78%) with a lower standard deviation (from 15.5% in *Conv5* to 11.7% in *Conv3* for sensitivity), and for accuracy (from 10.4% in *Conv5* to 6.8% in *Conv3*). We observe this reduction in the standard deviation on accuracies when comparing pressure-based images and raw images (from 10.8% for the best accuracy on raw images (*fc7*) to 6.8% for accuracy in *Conv3*). *Conv3* strikes a better balance between sensitivity and specificity metrics. For all these reasons, *Conv3* gives good results.

#### 3.1.2. Altitude-Based Hybrid Images

Table 3 shows that altitude-based hybrid images also improve considerably the results compared to raw images. With descriptors from *Conv3*, we obtain a significant increase in the sensitivity: from 62% on raw images (Table 1) to 81% on altitude images (Table 3). Especially, we note that the standard deviation of sensitivity decreases roughly by a factor of 3 (from 16% to 5.4%). We also note a similar accuracy to that obtained on pressure-based images, also in *Conv3*.

#### 3.1.3. Velocity-Based Hybrid Images

Table 4 shows that velocity hybrid images provide a significant improvement in performance compared to raw images (Table 1) in terms of sensitivity: from 62% to 72% with *fc7* descriptors. Moreover, although the accuracy is comparable (74.5% on raw images versus 75.5% in velocity-based images), its variance between the 10 samplings decreases significantly (from 10.8% on raw images with *fc7* descriptors, to 6.1% on velocity-based images with fc7 descriptors).

Again, *Conv3* descriptors give a better sensitivity than those from *fc7*. However, in this case, *fc7* descriptors remain the best in terms of accuracy.

#### 3.1.4. Acceleration-Based Hybrid Images

Table 5 displays performance measures obtained on acceleration-based images. We observe lower sensitivity values compared to images embedding other dynamic parameters. Moreover, the best accuracy, obtained on *Conv5* descriptors, is equivalent to that obtained on raw images (with *fc*7 in Table 1).

In the following, we study the fusion of different SVM experts’ decisions with a majority voting scheme.

### 3.2. Fusion of Expert’s Decisions in Each Layer

We analyze the impact of fusing classifiers’ decisions when considering input feature descriptors from different layers of AlexNet.

Table 6 displays the results obtained when combining the decisions of experts trained on feature descriptors issued from pressure-based, altitude-based, and velocity-based images. Table 7 displays those combining the decisions of experts trained on feature descriptors issued from pressure-based, altitude-based, and acceleration-based images.

In Table 6, we observe that when considering *Conv3* descriptors, decision fusion improves significantly classification performance: the accuracy and the sensitivity reach 81.5% and 84%, respectively, outperforming all the previous results. With respect to raw images (Table 1), there is an absolute improvement in sensitivity of 22%, confirming the power of our approach. We note that accuracy and sensitivity both increase when extracting descriptors in the first layers, until reaching *Conv3*; in higher-level representations, instead, accuracy and sensitivity decrease.

Results in Table 7 also are the best for *Conv3* descriptors, showing the same specificity (79%) as in Table 6, but lower sensitivity and accuracy on average over the 10 samplings. Actually, acceleration-based images led to a lower sensitivity (71%), as shown in Table 5.

### 3.3. Other Decision Fusion Schemes

Globally, in previous results, we remark a trend: high-level features such as *fc7* and *Conv5* lead to higher accuracies and specificities, but since *Conv2* performance is already satisfactory with a good balance between specificity and sensitivity.

For a better insight into this trend, for the best combination of dynamic parameters (pressure, altitude, velocity), we compare fusing the decisions of SVM experts trained on lower layers’ descriptors (*Conv2*, *Conv3*) and *fc7*, with the case in which SVM experts are trained on higher layers’ descriptors (*Conv3*, *Conv5*) and *fc7*. We report the results respectively in Table 8 and Table 9.

We first study these fusion schemes per parameter, before considering all dynamic parameters altogether in both Table 8 and Table 9 (case combining pressure, altitude, and velocity parameters, denoted as (P, Alt, and V)). When studying fusion for a single dynamic parameter, we fuse the decisions of only three experts (each trained on descriptors extracted at one layer of AlexNet); by extension, when combining three dynamic parameters, we fuse the decisions of nine SVM experts (descriptors extracted at three different levels of representation for each dynamic parameter).

In Table 8, no improvement is observed with this decision fusion scheme on *(P,Alt,V)* compared to the best result in Table 6, when fusing the decisions of experts trained on pressure, altitude, and velocity descriptors, but extracted at a single layer (*Conv3*). This result highlights that *Conv3* extracts the most pertinent features for our classification task.

Table 9 reports the same experiment on decision fusion when considering dynamic representations from higher levels of AlexNet (*Conv3*, *Conv5*, and *fc7*). We note that fusing decisions of experts trained on higher-level descriptors strongly degrades the average sensitivity (from 84% in Table 6 to 76% in Table 9). This confirms the trend mentioned at the beginning of Section 3.3.

### 3.4. Comparison to Low-Level Fusion of Hybrid Images

In previous sections, we studied decisions’ fusion of different experts trained on AlexNet descriptors extracted at different levels of representation. In this section, our aim is to assess the scope of low-level fusion of hybrid pressure, velocity, and altitude images.

We present as input to AlexNet each type of hybrid image through one channel. Then, we extract descriptors at different layers by transfer learning, as explained in Section 3.1. Following the same protocol of 10 random samplings described in Section 2.2.3, we train one SVM expert on a feature vector extracted at each layer. Table 10 reports the obtained results for all layers.

We remark that this low-level fusion scheme (Table 10) clearly gives lower performance than decision fusion schemes considering the same dynamic information (Table 6). The best descriptors, found in the fourth convolutional layer, lead to lower average accuracy (from 81.5% in Table 6 to 77.5% in Table 10) and especially lower average sensitivity (from 84% in Table 6 to 76% in Table 10). Moreover, we observe that the standard deviations of such measures increase significantly with low-level fusion. These results reveal that the process of automatic feature extraction is effective when considering only one type of hybrid image as input to the deep network.

## 4. Discussion and Conclusions

The present study proposes a novel scheme for the automatic assessment of early-stage AD, based on Archimedes’ spiral. It exploits transfer learning for feature extraction on “hybrid” spiral images, which convey dynamic information of the handwritten gesture. Such images, generated from online sequences captured by a digitizer, embed in the spiral trajectory image, in a pointwise manner, dynamic information encoded in greyscale. We consider four types of hybrid images, embedding separately pressure, altitude, velocity, and acceleration values in the spiral trajectory, both when the pen is on paper and in-air.

Our experiments demonstrate that this hybrid encoding of the spiral gesture leads to more discriminant features, extracted by transfer learning, compared to those obtained on raw spiral images that only convey spatial information of the trajectory. We actually assess the discriminant power of such features with the accuracy, sensitivity, and specificity metrics obtained after training an SVM classifier on feature vectors extracted from different layers of AlexNet.

When embedding dynamic parameters in the spiral trajectory, we first remark a strong improvement of the average sensitivity, from 62% on the raw image (Table 1), to 77% with pressure (Table 2), to 81% with altitude (Table 3), to 76% with velocity (Table 4), and to 71% with acceleration (Table 5). Our methodology coupling hybrid images with transfer learning thus enhances the detection of AD at an early stage. Moreover, our methodology leads to more balanced average sensitivity and specificity values: on raw images, we note a difference of 25% between specificity (87%) and sensitivity (62%), while on hybrid images such difference is significantly reduced, as it appears in Table 2, Table 3, Table 4 and Table 5. This means that our approach enhances significantly sensitivity while maintaining high specificity values.

Our work also shows that such improvements required studying representations extracted by the network at different layers. Indeed, on raw images, *fc*7 gives the best accuracy, with high specificity at the price of a very low sensitivity. For hybrid images, in general, we obtain instead *Conv3* discriminant descriptors that strike a better balance between sensitivity and specificity: it is the case for pressure, altitude, and velocity images. For acceleration, we obtain the best performance at *Conv5*.

Concerning dynamic parameters, pressure, altitude, and velocity lead to more discriminant features between AD and HC, comparatively to acceleration. Actually, acceleration leads to the lowest average accuracy (74.5%), the same obtained on raw images, but with a better balance between sensitivity and specificity. The highest average accuracies are obtained with pressure images (77% in *Conv3* and 79.5% in *Conv5*) and altitude (77.5% in *Conv3*), followed by velocity images (73.5% in *Conv3*). This confirms our previous result in [28], obtained on the same population, but on signature samples: pressure and altitude convey a high discriminant power for early AD detection. More precisely, the altitude angle leads to the highest sensitivity (81%), highlighting that the way the pen is held by the writer during the spiral gesture, is of significant importance for AD detection. This fact has also an impact on pen pressure, explaining why pressure images also show a high sensitivity (78%).

We confirm these results when fusing by majority voting the decisions of experts trained on descriptors extracted from AlexNet. Given our previous analysis of pressure and altitude descriptors, we compare two configurations both including such descriptors: one fusing decisions of experts trained on pressure, altitude and velocity descriptors, and another fusing decisions of experts trained on pressure, altitude, and acceleration descriptors. The first outperforms all previous results: the accuracy reaches 81.5% on average and sensitivity increases to 84%, also reducing the standard deviation over the 10 samplings (Table 6). Compared to the classification performance obtained on descriptors from raw images, there is an absolute improvement in sensitivity of 22% (from 62% in Table 1 to 84% in Table 6), and in accuracy of 7%, confirming the power of our approach. This result, which is the best, is obtained with intermediate-level descriptors from the third convolutional layer (*Conv3*), confirming the trend already observed in most types of hybrid images.

Moreover, we note that accuracy and sensitivity both increase when extracting descriptors in the first layers, since *Conv2*; instead, on descriptors of higher layers, accuracy and sensitivity decrease. For this reason, we studied majority voting with more experts, each trained respectively on descriptors from *fc7* and lower layers, such as *Conv2* and *Conv3*, or on higher ones, such as *Conv3* and *Conv5*. None of such fusion schemes improves classification results, leading to the conclusion that *Conv3* already extracts the most discriminant descriptors for our specific classification task on spiral images.

Finally, decision fusion outperforms low-level fusion, namely when giving as input to AlexNet each type of hybrid image through one channel and extracting descriptors at different layers. Low-level fusion of the best combination of hybrid images (pressure, velocity, and altitude) led indeed to lower accuracy (77.5% in Table 10 versus 81.5% in Table 6) and lower sensitivity (76% in Table 10 versus 84% in Table 6), with a larger standard deviation over the 10 samplings. Automatic feature extraction is indeed efficient when considering as input to AlexNet only one type of hybrid image.

With regard to the literature on degenerative diseases, most works (on Parkinson’s disease) exploit decision fusion successfully, but on several tasks [16,17,18,20,21]. Our work focuses on one task in order to show its intrinsic potential for AD detection. This study demonstrates that the digitized spiral task can be used successfully in a standalone mode for early AD detection, with a precise transfer learning methodology.

Furthermore, our methodology allows confirming observed trends in our previous works about the most pertinent dynamic parameters for AD detection: altitude, pen pressure, and velocity convey precious information on the wrist-hand-finger system for pathology detection. The best representations for dynamic information can be extracted by AlexNet automatically at *Conv3*, and decision fusion is the most reliable paradigm for discriminating between the two populations. The high performance obtained on the spiral with this fusion scheme makes it possible to consider other short tasks as signatures or other graphomotor gestures for improving detection.

In the future, we aim at studying transfer learning based on other DL models following the same methodology. The objective is to analyze the effectiveness of descriptors extracted from more complex architectures compared to those obtained with AlexNet. Moreover, we will go further in studying other strategies for encoding dynamic information in visual contents, in order to enhance the benefit of exploiting transfer learning.

## Figures and Tables

**Table 1:** Performance (in %) on raw images with descriptors from all layers.

Layer	Accuracy	Sensitivity	Specificity
Conv1	63.0 ± 9.0	69.0 ± 21.7	57.0 ± 27.6
Conv2	68.5 ± 10.5	72.0 ± 12.5	65.0 ± 16.3
Conv3	65.5 ± 11.5	67.0 ± 11.0	64.0 ± 23.3
Conv4	67.5 ± 8.7	65.0 ± 20.6	70.0 ± 21.3
Conv5	71.0 ± 8.9	68.0 ± 17.2	74.0 ± 27.6
**fc7** ^1^	**74.5 ± 10.8**	**62.0 ± 16.0**	**87.0 ± 14.2**

^1^ Best case.

**Table 2:** Performance (in %) on pressure-based images with descriptors from all layers.

Layer	Accuracy	Sensitivity	Specificity
Conv1	71.0 ± 4.4	48.0 ± 9.8	94.0 ± 4.9
Conv2	74.0 ± 9.4	72.0 ± 14.0	76.0 ± 14.3
**Conv3**	**77.0 ± 6.8**	**78.0 ± 11.7**	**76.0 ± 14.3**
Conv4	77.0 ± 11.0	75.0 ± 12.0	79.0 ± 13.7
**Conv5** ^1^	**79.5** ± **10.4**	**77.0** ± **15.5**	**82.0** ± **4.9**
fc7	77.5 ± 6.4	74.0 ± 12.0	81.0 ± 7.0

^1^ Best case.

**Table 3:** Performance (in %) on altitude-based images with descriptors from all layers.

Layer	Accuracy	Sensitivity	Specificity
Conv1	69.0 ± 9.3	67.0 ± 11.9	72.0 ± 18.9
Conv2	75.5 ± 11.5	75.0 ± 15.0	76.0 ± 21.5
**Conv3** ^1^	**77.5 ± 10.5**	**81.0 ± 5.4**	**74.0 ± 21.5**
Conv4	74.0 ± 10.7	77.0 ± 7.8	71.0 ± 20.7
Conv5	69.0 ± 9.2	70.0 ± 18.4	68.0 ± 18.9
fc7	71.0 ± 7.0	71.0 ± 13.7	71.0 ± 18.7

^1^ Best case.

**Table 4:** Performance (in %) on velocity-based images with descriptors from all layers.

Layer	Accuracy	Sensitivity	Specificity
Conv1	65.0 ± 6.7	73.0 ± 17.9	57.0 ± 15.5
Conv2	71.0 ± 9.4	74.0 ± 10.2	68.0 ± 14.0
Conv3	73.5 ± 5.9	76.0 ± 11.1	71.0 ± 12.2
Conv4	73.0 ± 4.0	76.0 ± 13.6	70.0 ± 12.6
Conv5	75.5 ± 7.6	68.0 ± 15.4	83.0 ± 15.5
**fc7** ^1^	**75.5** ± **6.1**	**72.0** ± **13.3**	**79.0** ± **13.0**

^1^ Best case.

**Table 5:** Performance (in %) on acceleration-based images with descriptors from all layers.

Layer	Accuracy	Sensitivity	Specificity
Conv1	65.5 ± 12.7	47.0 ± 32.3	84.0 ± 14.3
Conv2	72.5 ± 7.5	69.0 ± 13.0	76.0 ± 19.1
Conv3	72.0 ± 6.8	67.0 ± 13.5	77.0 ± 14.2
Conv4	68.0 ± 8.7	70.0 ± 20.5	66.0 ± 21.5
**Conv5** ^1^	**74.5 ± 8.5**	**71.0 ± 19.2**	**78.0 ± 14.3**
fc7	70.5 ± 6.9	66.0 ± 16.9	75.0 ± 14.3

^1^ Best case.

**Table 6:** Fusion of SVM experts’ decisions trained on pressure, altitude, and velocity descriptors extracted from all layers.

Layer	Accuracy	Sensitivity	Specificity
Conv1	74.5 ± 9.9	70.0 ± 11.8	79.0 ± 17.0
Conv2	79.5 ± 9.6	81.0 ± 5.4	78.0 ± 16.6
**Conv3** ^1^	**81.5 ± 7.1**	**84.0 ± 6.6**	**79.0 ± 13.7**
Conv4	76.5 ± 6.3	77.0 ± 10.0	76.0 ± 12.8
Conv5	77.0 ± 8.4	74.0 ± 12.8	80.0 ± 17.0
fc7	77.5 ± 5.1	74.0 ± 9.2	81.0 ± 11.4

^1^ Best case.

**Table 7:** Fusion of SVM experts’ decisions trained on pressure, altitude, and acceleration descriptors extracted from all layers.

Layer	Accuracy	Sensitivity	Specificity
Conv1	74.0 ± 7.7	60.0 ± 17.9	88.0 ± 10.8
Conv2	79.5 ± 7.6	81.0 ± 12.2	78.0 ± 16.6
**Conv3** ^1^	**80.5 ± 6.1**	**82.0 ± 7.5**	**79.0 ± 13.7**
Conv4	74.0 ± 9.2	74.0 ± 10.2	74.0 ± 19.1
Conv5	76.0 ± 7.3	74.0 ± 13.6	78.0 ± 10.8
fc7	75.0 ± 4.5	73.0 ± 12.7	77.0 ± 11.0

^1^ Best case.

**Table 8:** Fusion of experts’ decisions fed with dynamic representations from Conv2, Conv3, and fc7.

Dynamic Representations	Accuracy	Sensitivity	Specificity
P (Pressure)	77.0 ± 9.5	77.0 ± 14.2	77.0 ± 14.2
Alt (Altitude)	77.5 ± 11.0	82.0 ± 4.0	73.0 ± 22.4
V (Velocity)	74.5 ± 5.4	77.0 ± 9.0	71.0 ± 12.2
**(P, Alt, V)** ^1^	**81.0 ± 7.7**	**84.0 ± 6.6**	**78.0 ± 14.7**

^1^ Best case.

**Table 9:** Fusion of experts’ decisions fed with dynamic representations from Conv3, Conv5, and fc7.

Dynamic Representations	Accuracy	Sensitivity	Specificity
P (Pressure)	80.5 ± 8.8	77.0 ± 14.9	84.0 ± 15.6
Alt (Altitude)	73.0 ± 10.0	74.0 ± 15.0	72.0 ± 22.7
V (Velocity)	75.0 ± 5.9	70.0 ± 11.8	80.0 ± 13.4
**(P, Alt, V)** ^1^	**79.0 ± 7.0**	**76.0 ± 11.1**	**82.0 ± 16.6**

^1^ Best case.

**Table 10:** Classification results (in %) after low-level fusion of pressure, altitude, and velocity hybrid images in AlexNet input channels.

Layer	Accuracy	Sensitivity	Specificity
Conv1	73.5 ± 11.2	68.0 ± 19.9	79.0 ± 17.6
Conv2	77.0 ± 12.9	71.0 ± 19.7	83.0 ± 17.3
Conv3	76.5 ± 12.5	69.0 ± 23.9	84.0 ± 15.6
**Conv4** ^1^	**77.5 ± 12.5**	**76.0 ± 15.6**	**79.0 ± 17.0**
Conv5	75.5 ± 7.6	66.0 ± 18.5	85.0 ± 17.6
fc7	75.0 ± 8.7	62.0 ± 18.3	88.0 ± 8.7

^1^ Best case.

## Data Availability

We conducted the study on a private dataset acquired at Broca Hospital in Paris, in the framework of the ALWRITE project, which is a French research project on handwriting analysis for early AD assessment. This study was approved by the Ethics Committee “Ile-de-France III” of APHP (Assistance Publique Hôpitaux de Paris). All participants freely signed a consent form after receiving information on the study. There are legal and ethical restrictions on sharing these data. We did not have permission to share these data publicly.
